# Research on the Methods for the Mass Production of Multi-Scale Organs-On-Chips

**DOI:** 10.3390/polym10111238

**Published:** 2018-11-07

**Authors:** Andrés Díaz Lantada, Wilhelm Pfleging, Heino Besser, Markus Guttmann, Markus Wissmann, Klaus Plewa, Peter Smyrek, Volker Piotter, Josefa Predestinación García-Ruíz

**Affiliations:** 1 Product Development Laboratory, Mechanical Engineering Department, Universidad Politécnica de Madrid (UPM), c/ José Gutiérrez Abascal 2, 28006 Madrid, Spain; 2 Institute of Applied Materials—Applied Materials Physics, Karlsruhe Institute of Technology (KIT), Hermann-von-Helmholtz-Platz 1, 76344 Eggenstein-Leopoldshafen, Germany; wilhelm.pfleging@kit.edu (W.P.); heino.besser@kit.edu (H.B.); peter.smyrek@kit.edu (P.S.); 3 Karlsruhe Nano Micro Facility (KNMF—Helmholtz Research Infrastructure), Hermann-von-Helmholtz-Platz 1, 76344 Eggenstein-Leopoldshafen, Germany; markus.guttmann@kit.edu (M.G.); markus.wissmann@kit.edu (M.W.); klaus.plewa@kit.edu (K.P.); volker.piotter@kit.edu (V.P.); 4 Institute of Microstructure Technology (IMT), Karlsruhe Institute of Technology (KIT), Hermann-von-Helmholtz-Platz 1, 76344 Eggenstein-Leopoldshafen, Germany; 5 Institute of Applied Materials, Materials Process Technology, Karlsruhe Institute of Technology (KIT), Hermann-von-Helmholtz-Platz 1, 76344 Eggenstein-Leopoldshafen, Germany; 6 Departamento de Biología Molecular, Universidad Autónoma de Madrid, 28049 Cantoblanco-Madrid, Spain; josefap.garcia@uam.es

**Keywords:** organs-on-chips, labs-on-chips, additive manufacturing, laser materials processing, electroforming, mold fabrication, micro-injection molding, mass production, biomedical microdevices

## Abstract

The success of labs- and organs-on-chips as transformative technologies in the biomedical arena relies on our capacity of solving some current challenges related to their design, modeling, manufacturability, and usability. Among present needs for the industrial scalability and impact promotion of these bio-devices, their sustainable mass production constitutes a breakthrough for reaching the desired level of repeatability in systematic testing procedures based on labs- and organs-on-chips. The use of adequate biomaterials for cell-culture processes and the achievement of the multi-scale features required, for in vitro modeling the physiological interactions among cells, tissues, and organoids, which prove to be demanding requirements in terms of production. This study presents an innovative synergistic combination of technologies, including: laser stereolithography, laser material processing on micro-scale, electroforming, and micro-injection molding, which enables the rapid creation of multi-scale mold cavities for the industrial production of labs- and organs-on-chips using thermoplastics apt for in vitro testing. The procedure is validated by the design, rapid prototyping, mass production, and preliminary testing with human mesenchymal stem cells of a conceptual multi-organ-on-chip platform, which is conceived for future studies linked to modeling cell-to-cell communication, understanding cell-material interactions, and studying metastatic processes.

## 1. Introduction

The advent of labs- and organs-on-chips, thanks to joint advances in design techniques, materials science, micro-manufacturing technologies, and cell-culture processes, is providing researchers with new ways of modeling and studying disease, while helping to develop related therapies in a non-invasive, sustainable, and quite straightforward approach [1,2,3,4]. Within these labs-and organs-on-chips cells grow, move, communicate, and interact with their companions in much more physiological and biomimetic conditions than when cultured upon conventional Petri dishes.

Even if some key aspects, such as obtaining a real three-dimensional representation of the tissues and organs being studied or promoting in vitro vascularization, are still unsolved, these microsystems are already having a relevant impact in biomedical research. Foundational experiences in this field resorted to the use of UV-lithography and etching upon silicon wafers, following procedures from the microelectronics industry [5], although the biochemical and biomechanical interactions between cells and silicon does not always provide the desired level of biomimicry. The development and application of soft-lithographic procedures, to achieve polymeric (mainly polydimethylsiloxane (PDMS) but sometimes even paper) parts, provided a new set of resources for researchers and for the rapid manufacture of labs- and organs-on-chips [6,7]. 

More recently, progresses in additive manufacturing technologies and developments linked to the materials abilities or characteristics for being additively processed have also made an impact in the biomedical engineering arena and, especially, in the development of labs- and organs-on-chips [8,9,10]. These additive manufacturing technologies provide designers with an increased level of geometrical complexity and flexibility, which is necessary for devices trying to imitate the intricate shapes of the human body, organs, and tissues. In addition, additive manufacturing technologies also promote the level of integration and even monolithic labs- and organs-on-chips have been recently obtained, which constitutes and advance, in terms of ergonomics, for researchers usually having to deal with several parts and devices in their labs. 

However, in many cases, the required level of precision for interacting at single cellular level is difficult to achieve; hence alternative techniques or multi-scale approaches are required. Despite the aforementioned advances, the success of labs- and organs-on-chips as transformative technologies in biomedical engineering still depends on our ability of solving some current challenges related to their design, modeling, manufacturability, and usability. Among present needs for the industrial scalability and impact promotion of these bio-devices, their sustainable mass production constitutes a breakthrough for reaching the desired level of repeatability in systematic testing procedures based on labs- and organs-on-chips. The use of adequate biomaterials for cell-culture processes and the achievement of the multi-scale features required, for in vitro modeling the physiological interactions among cells, tissues, and organoids, prove to be demanding requirements in terms of production. 

This study presents an innovative synergistic combination of technologies, including: laser stereolithography, laser materials processing on micrometer scale, electroforming, and micro-injection molding, which enables the rapid creation of 3D multi-scale mold cavities for the industrialized mass production of labs- and organs-on-chips using thermoplastics apt for in vitro testing. The procedure is validated by the design, rapid prototyping, mass production, and preliminary testing with human mesenchymal stem cells (hMSCs) of a conceptual multi-organ-on-chip platform, which is conceived for future studies linked to modeling cell-to-cell communication, cell-material interactions, and metastatic processes. Current research works upon initial trials developed by our team [11,12], but achieves here more precision, a larger final size of the mold inserts and micro-injected replicas and an increased degree of complexity, including multiple organ chambers and biomimetic features for an improved performance. At the same time, a more complete validation of the developed multi-organ-on-chip, by testing with hMSCs, is presented and analyzed. The biological testing stands out by the employment of a human mesenchymal stem cell (h-MSC) conditioned medium (CM), which is a solution of growth factors generated progenitor stem cells themselves, that helps to enhance the microsystem’s response by promoting cell adhesion, proliferation, and final long-term viability in the cell-culture trials. 

The relevant advantages of the employed synergistic combination of technologies, especially in terms of multi-scale geometrical freedom, structure accuracy, use of industry-capable methods and productivity, towards mass-manufacture of polymeric labs- and organs-on-chips for increased industrial and social impact are finally discussed, considering also the preliminary assessment of material’s viability achieved thanks to the cell-culture tests performed.

## 2. Materials and Methods 

### 2.1. Design Process

The current development of biomedical microdevices is very often supported by a wide set of available computer-aided design, engineering, and manufacturing software programs, which help with the straightforward definition of geometries and with the creation of prototypes for systematic testing. In present study, the geometry of the multi-chamber (lab-/organ-)on-a-chip is designed with the help of NX-8.5 (Siemens PLM Solutions, purchased from Avantek, Spain). The design is oriented towards a single step manufacture of a master model, to be subsequently improved by micro-machining techniques and transformed into a mold insert or cavity for mass production. We consider from the design stage the particular aspects of the production processes (as described in the following paragraphs) for obtaining computer-aided design (CAD) model-oriented manufacturing. 

**Figure 1:**
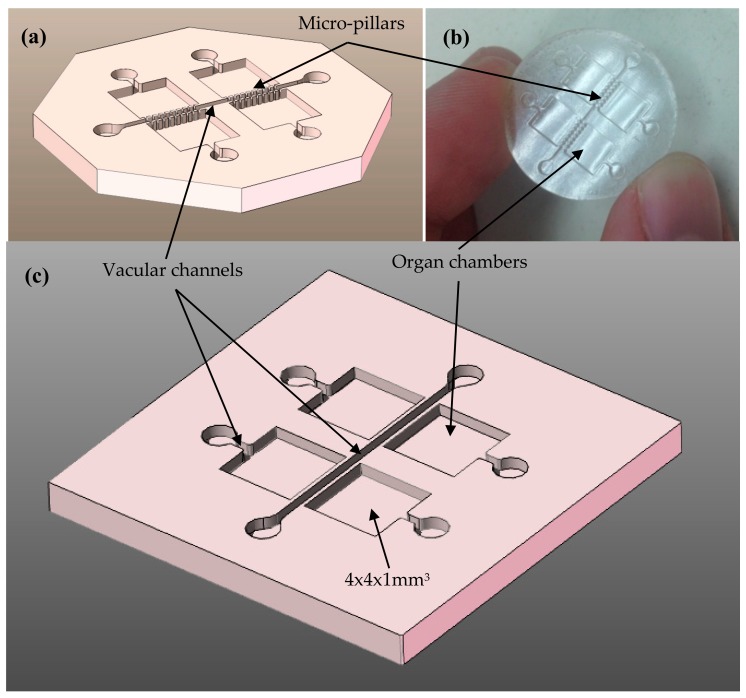
Computer-aided design CAD and conceptual prototype of a multi-organ-on-chip platform: (**a**) Computer-aided design showing inlets, vascular channels, and organ chambers. (**b**) Preliminary test obtained using laser stereolithography in epoxy resin (lack of precision). (**c**) Modified computer-aided design for generation of connecting “gates” by laser materials processing of the walls separating the vascular channels and the organ chambers.

Figure 1 shows schematically the computer-aided design of the proposed multi-purpose biomedical microdevice, in fact a lab-on-chip system or organ-on-chip platform, aimed at improved cell co-culture strategies. The inlets are thought for enabling the incorporation of different cell types and growth of trophic factors (for instance varied parenchymal cells for modeling different tissues, endothelial cells for modeling vasculature, pericytes for analyzing self-healing strategies, vascular endothelial growth factor “VEGF” and other trophic or growth factors), which would grow, move and colonize the microsystem, while interacting with their companions within physiologically biomimetic and controlled micro-environments for enhanced modeling of disease processes and related therapies or environmental influences. The organ chambers and the vascular channels are connected by 200–250 µm-wide openings in the separation walls, to allow cell-to-cell interactions and to prevent massive migrations from the organ chambers to the vascular channels and vice versa. Figure 1a presents the original concept with the micro-pillars separating the organ chambers and the vascular channels and Figure 1b illustrates an initial attempt to obtain the proposed concept by laser stereolithography as rapid prototyping technology (see also Section 2.2). Being true that a complete system would involve an additional bonded closing cover and possibly inlet and outlet channels for circulating fluids, in this study we focus on the generation of the multi-channel and multi-chamber layer, which supports cell placement and subsequent pattern formation and constitutes the most challenging component in terms of micro-manufacturing, as closing covers typically involve just the machining of inlet and outlet holes.

However, the initial prototype from Figure 1b and an additional set of prototypes, with systematic variations in the distances between the micro-pillars, let us understand that an extra degree of precision is required for achieving the separation micro-pillars with the desired functionality and geometrical fidelity. In the case of conventional laser stereolithography, the micro-pillars obtained are either too separated for limiting massive migrations or completely joined due to a limited accuracy of the additive photopolymerization process. Consequently, the design is modified to that shown in Figure 1c, in which continuous walls separate the “organ” chambers and “vascular” channels. As described in Section 2.2, such walls are to be micro-drilled with more precise micro-manufacturing techniques, i.e., laser materials processing, after prototyping by laser stereolithography, to generate the inter-connecting gates between the organ chambers and the vascular channels. The final CAD features are designed, taking into account the geometrical limitations and precision of the first-stage prototyping process, which leads to the master prototypes of green parts. Features down to 300–350 µm are designed, with 300–350 µm-wide vascular channels and with organ chambers of 4 × 4 × 1 mm^3^. The whole multi-organ-on-chip can be circumscribed by a box of 30 × 30 × 3 mm^3^ (X, Y, Z, directions respectively). 

In our opinion, the conceived biomedical microdevice is quite a versatile one, as may be linked to a wide set of lab-on-chip and organ-on-chip biomedical applications, although the initial concept is just aimed at studying cell-to-cell communication, cell-material interactions, cell dynamics and metastatic processes. For instance, the separation by micro-pillars between organ chambers and vascular channels, may promote cell co-culture applications aimed at reproducing the physiological interactions of different cells within human organs in a biomimetic way. 

This separation approach, which does not rely on the use of multi-layers divided by micro-porous membranes, may help to promote production due to having just one active layer in the microsystem and may minimize leakage and other problems during lab testing. The circular inlets and outlets can be also employed for the generation of dynamic cell-culture conditions by connecting to adequate tubing and pumping systems, which can be of interest for driving mesenchymal cells towards gene expression and differentiation into different tissues, as well as for enhancing the generation of vasculatures within these types of in vitro models. Visualization and imaging tasks upon the different organ chambers and focusing on the interactions between the parenchymal tissues and the vasculature may be also performed more clearly. 

### 2.2. Manufacturing Process

#### 2.2.1. Additive Generation of Master Prototypes or Initial “Green” Parts

Once obtained, the CAD model with the desired geometry is saved as a stl (standard tessellation language or stereolithography) file for further processing. The master models or “green parts”, similar to the model shown in Figure 1b, are manufactured using the SLA-3500 laser stereolithography machine developed and commercialized by 3D Systems (Three D Systems Circle, Rock Hill, SC 29730, USA). Epoxy photo-resin Accura60^®^, also commercialized by 3D Systems, is employed as prototyping material for the master prototypes or green parts. 

In short, the geometry present in the .stl file is “sliced” with the help of Lightyear software, provided with the stereolithography system, which generates the trajectories for the laser beam, which works layer-by-layer activating the polymerization of the resin and generates a three-dimensional (3D) solid component by this additive photopolymerization process. 

After manufacture, the master prototypes are cleaned by two-minute bath into a chemical solution of acetone and post-cured (to enhance mechanical properties) in an UV-oven for 20 min, according to resin’s specifications. Final polishing of the bottom surface helps to eliminate surface residuals of the supporting structures typical of liquid-based additive manufacturing techniques. The upper surface is left unpolished for avoiding and dimensional accuracy changes in the relevant surface of the part and because of its adequate mirror-like quality.

#### 2.2.2. Laser Materials Processing of Master Prototypes

Despite several technologies being available for the micro-manufacture of 2.5D and 3D geometries for biomedical microdevices, one of the most relevant among subtractive micro-machining technologies is laser ablation, as it can work with a wide set of materials and help manufacturers to achieve complex geometries. Among the main advantages of the process it is important to highlight the chemical- and contact-free process, the simple automation and the minimal heating and damage to surrounding zones of the processed material, which is quite important for polymers and polymer-matrix composites and important for metal materials. In principle, the lower the “HAZ” or heat-affected zone, the higher the achieved structure and surface quality, as less debris is generated and fewer phase changes are induced. Additionally, the possibility of automating the laser movement allows for the creation of complex geometries. Even thermal laser processes for industrial application became recently possible by introducing ultrafast laser micro-machining [13]. In this study, we combine laser ablation with the previously additively generated polymeric rapid prototypes or master models, to achieve the desired multi-scale manufacturing approach. The global structure is hence additively manufactured by laser stereolithography and, in a subsequent step, laser ablation is used for the generation of micro-gates, in the walls that separate the different channels.

**Figure 2:**
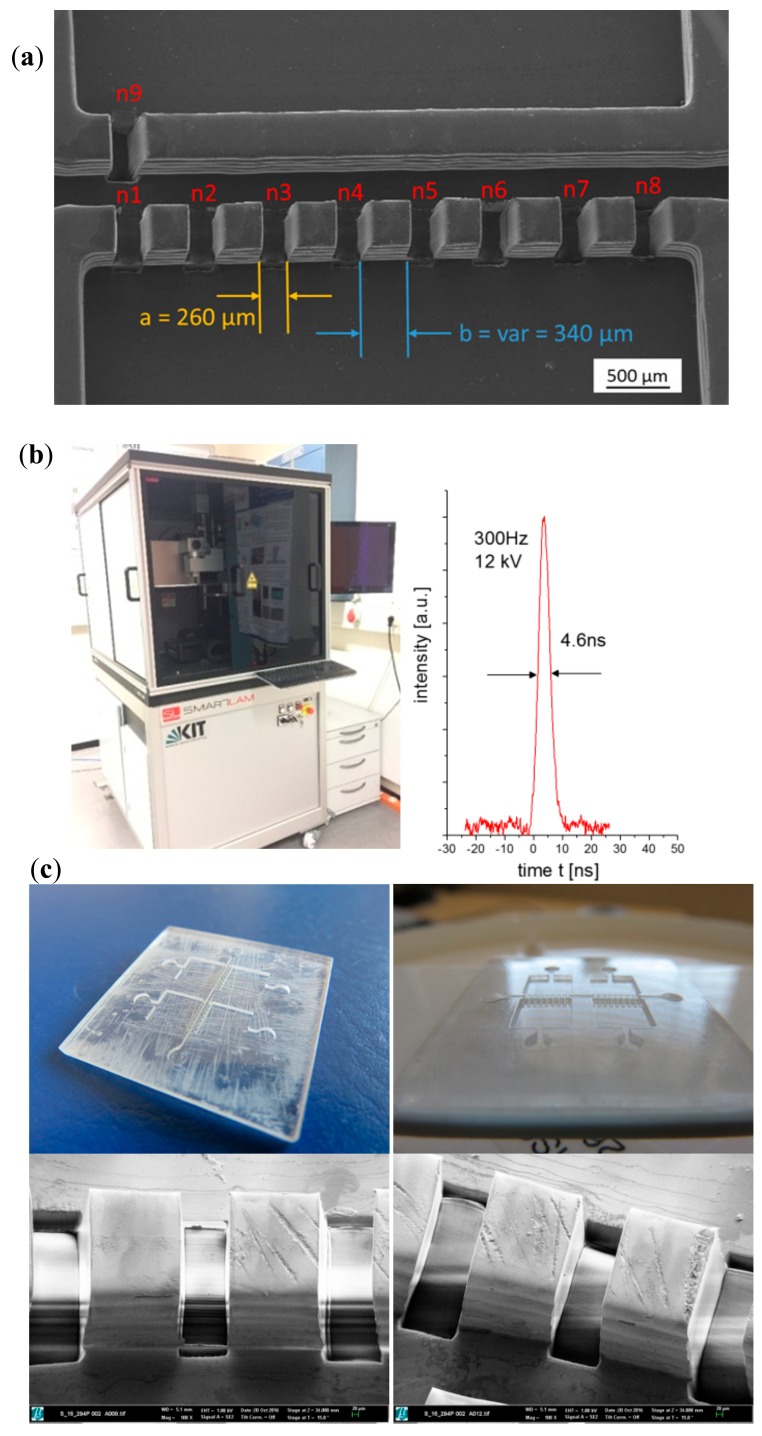
Laser materials processing performed upon the master epoxy resin prototypes for generating the connecting gates between vascular channels and organ chamber: (**a**) Dimensional overview of chambers, channels and connecting gates. (**b**) ArF-excimer laser micro-machining system and temporal laser pulse (right). (**c**) Digital and SEM images of the polymeric master after laser material processing. Details of the surface finish after laser materials processing (using a laser wavelength 193 nm, a laser fluence of 1.8 J/cm^2^ and a laser repetition rate 100 Hz).

First of all, the procedure is fine-tuned by performing preliminary tests upon flat samples, as presented in Figure 2. For this purpose, the appropriate laser process parameters (laser wavelength, laser pulse length, laser fluence, laser repetition rate, processing gas) must be identified regarding the laser materials interaction characteristics including laser absorption, ablation rate, debris formation and thermal impact to the surrounding material. For polymers, laser ablation depends on the absorptivity and thermal diffusivity of the materials. Laser ablation normally is related to a strong absorption of laser photons by the sample material, which means that the laser’s wavelength must be selected accordingly, to maximize absorption. With ultra-short laser pulses, however, ablation may take place therefore from multi-photon absorption at high peak intensities; hence also transparent objects and materials can be machined.

Once understood how the material interacts with laser radiation under defined processing conditions, additional operations linked to the desired final object can be carried out, even in a programmed way for periodic operations and patterns, as presented in Figure 2. Main results from laser ablation upon one of the epoxy master models or prototypes can be seen: 

The desired connections between channels and chambers of the multi-organ-on-chip microsystem, are presented and details down to 50 µm with very vertical walls and remarkable surface quality, which is maintained in the subsequent processing steps, as we discuss further on, are clearly appreciated in the prototype mold masters. 

For this type of laser structuring a high repetition rate excimer laser micro-machining system (Figure 2b) operating at a wavelength of 193 nm with pulse duration of 4–5 ns is applied. The laser beam is projected through a motorized mask onto the work piece using a de-magnifying projection lens to generate the desired image and pattern. The motorized mask in combination with a synchronized laser scanning procedure is necessary to achieve well defined channel structures as shown in Figure 2. Even submicron features are possible [14].

#### 2.2.3. Electroforming and Mechanical Treatment for Mold Insert Fabrication

The micro-structured polymeric masters (size of microdevice: 25 × 25 × 1.5 mm^3^; structure depth ~0.5 mm) need to be directly transferred or converted into a metallic mold insert or mold cavity by electroforming, following a previously developed process by our team with some relevant modifications [15]. 

First, the master made in acrylic resin Accura60^®^ is glued onto a thick copper substrate (84 × 54 × 8 mm^3^). In an evaporation process, master and substrate get coated with superimposed layers of 7 nm chromium and 40 nm gold. The chromium layer serves as adhesive layer and the gold layer acts as conductive plating base. The metallic layers promote a precise galvanic metal deposition throughout the whole 2.5D micro-structures previously created by laser ablation upon the masters. The copper substrate is fixed to an ad hoc plating holder that is immersed into galvanic bath. The nickel electroplating system with a boric acid containing (chloride-free) nickel sulfamate electrolyte (*T* = 52°C, pH 3.4…3.6) was developed especially for the nickel electroforming of micro-structures at KIT [16,17]. The use of this electrolyte leads to matt, nearly stress-free, thick nickel layers up to 10 mm without any warpage [16,17].

**Figure 3:**
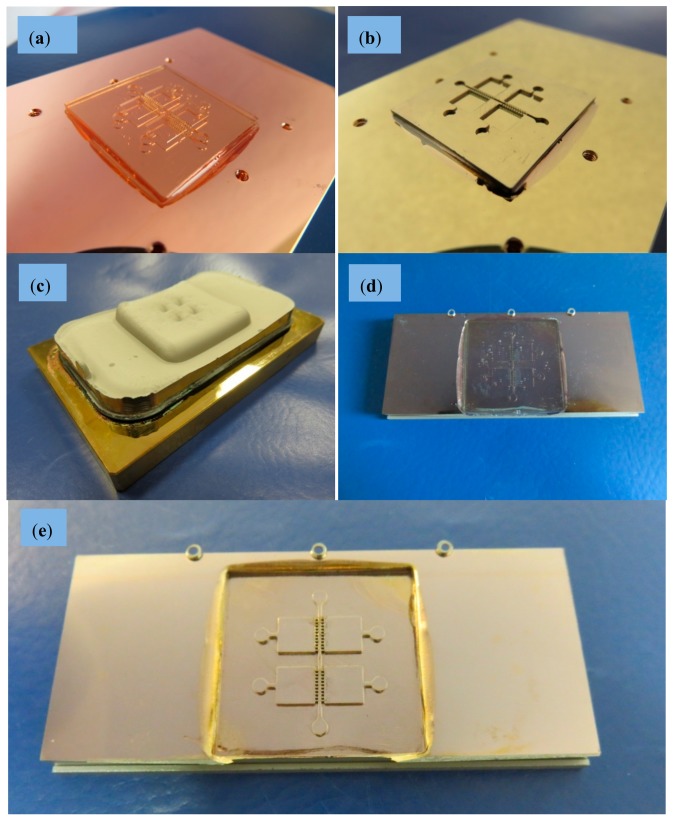
Nickel mold insert fabrication, different process steps: (**a**) Polymer master glued on copper substrate. (**b**) Substrate with glued master after Cr/Au metallization. (**c**) Substrate with 6 mm thick nickel block and overgrowth of the glued polymer master. (**d**) Nickel block inverted to a mold insert after wire-cut EDM and substrate removal. (**e**) Mold insert after wet-chemical master removal.

To promote an exact filling of the micro-structured geometries without defects, a slow growing process is planned and, accordingly, the current density is adjusted to 0.1 A/dm^2^ at the beginning of the plating process and is subsequently increased up to 1.5 A/dm^2^. Electroforming continues until a nickel layer with a thickness of 6 mm is achieved. To support the adhesion of the thick nickel block and to avoid a lift-off during the long plating time (>2 weeks), the copper substrate was equipped with six threaded holes for toothing. The overall procedure is presented in Figure 3.

**Figure 4:**
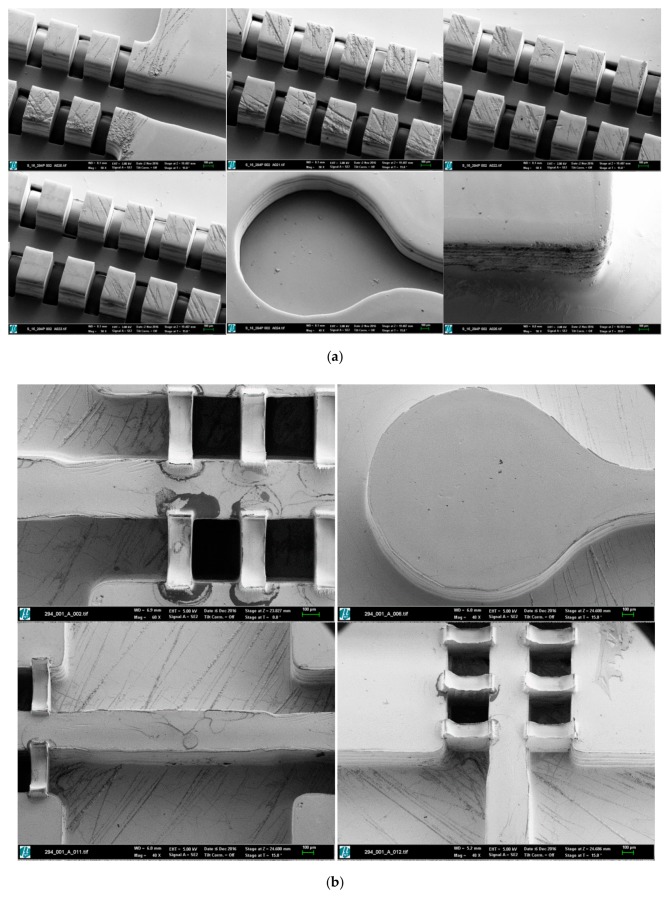
(**a**) SEM images of the master structures in Accura60^®^ after Cr/Au metallization. (**b**) SEM images of the nickel structures on the mold insert.

This electroplating process allows us to obtain a very stiff and homogenous metal block with a very uniform thickness, which is necessary for withstanding the mechanical and thermal stresses present in the injection molding process, used for creating the final biomedical microdevices in an industrial production way. The electroplated nickel block with flat surface and without any blowholes and dendrites is finally separated from the substrate and mechanically processed to the desired external dimensions (66 × 30 × 5 mm^3^) by wire-cut electro-discharge machining (EDM), so as to fit it into the available molding tool with interchangeable central zone for the final micro-injection molding process. The acrylic master is removed from the mold insert cavity by using a wet-chemical process and a specific cleaning agent with ultrasonic agitation at 80°C. A final rinsing completes the first part of nickel mold insert fabrication. Process characterization was supported by scanning electron microscopy (SEM) (see pictures provided in Figure 4 for details regarding precision of the process and surface quality).

Finally, to ensure that the bottom of the structure cavities is on the same height level as the outer unstructured areas of the mold insert (and to realize protruded metallic micro-structures) a final mechanical treatment of the insert was necessary. At first the structured area was protected by casting with poly(methyl methacrylate) (PMMA). Afterwards the thickness of the insert was reduced from 5 to 3 mm and the outer dimensions were adapted to 30 × 26 × 3 mm^3^ using a mechanical milling process. After final wet-chemical removal of the casting material the mold insert could be used for the following injection molding experiments.

The use of electroforming enables the direct galvanic replication of all structural details (in different scale from nano to micro) of the master. In addition, angled side walls can also be transferred from the polymeric master to the metallic mold insert. 

#### 2.2.4. Micro-Injection Molding of Final Parts

**Figure 5:**
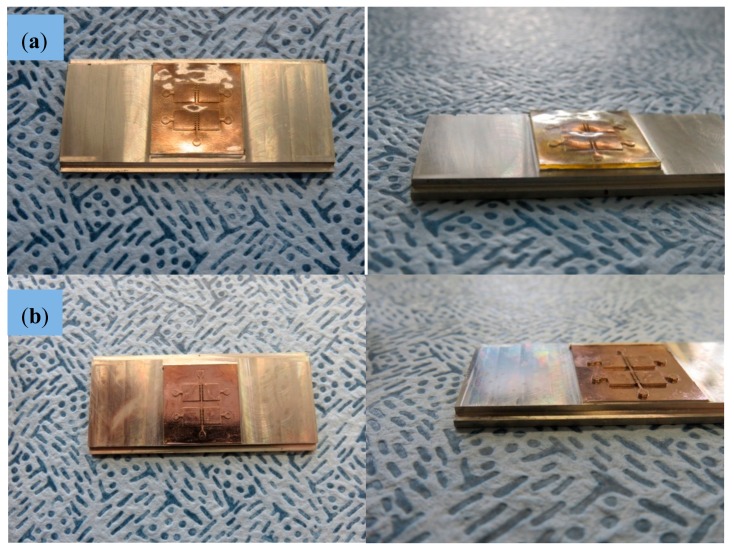
Nickel mold insert fabrication, different process steps: (**a**) Mold insert after mechanical milling process. (**b**) Mold insert after removal protecting layer ready for injection molding.

Before the injection trials can be performed, it is necessary to adjust the electroplated nickel mold inserts (Figure 5a) to one of the standard molding tools at KIT, as described in previous preliminary tests with some slight modifications [11]. As no further molding tool modification is needed the samples are replicated on a base plate, which forms the runner and gate system and acts as an auxiliary stabilizing feature for safe demolding. 

**Figure 6:**
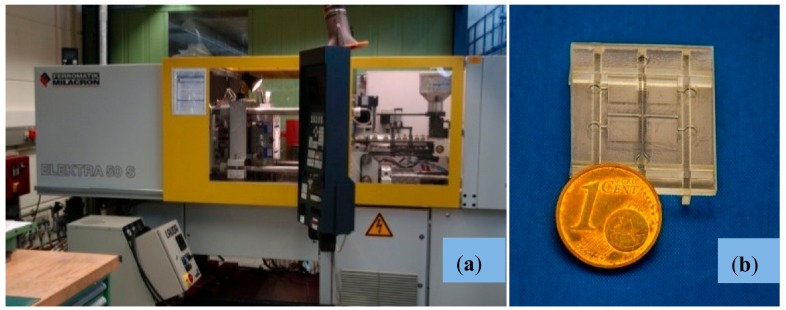
(**a**) Injection molding machine Ferromatik Electra 50S, especially equipped with tool evacuation and vario-thermal temperature control for replication of micro-structured parts. (**b**) Injection molded part (PMMA) after dismantling of auxiliary runner and base plate sections.

The replication trials are performed on a Ferromatik Elektra 50S injection molding machine (Figure 6a) which is equipped with advanced characteristics including tool evacuation and vario-thermal temperature control. The latter means that the core of the molding tool is heated up prior to the material injection for improved filling. After cavity filling, the tool core is cooled down to a temperature which guarantees safe demolding, which is required for not damaging the filigrane micro-structures of the base plate. 

This procedure allows for the replication of very detailed micro-structures with remarkable surface quality. For achieving the desired degree of precision with the micro-features present in the molds, the cycle times are significantly longer compared to isothermal process conduct and reached values of up to 300 s. It can be expected, however, that thorough optimization of the temperature control system under industrial conditions would result in much shorter times. 

Main injection molding parameters are included in Table 1 for informative purposes. Using these parameters and equipment 100 replicas of the biomedical microdevice are produced using PMMA, as an interesting thermoplastic material for the biomedical field. The particular PMMA type used for the study is Degalan G7E formerly distributed by Evonik Industries AG, Germany. An example of injection molded part ready for being tested for cell compatibility is shown in Figure 6b. The molds are not damaged during the manufacture and several additional replicas can be produced, if needed, for systematic testing or alternative applications. Initial dimensional measurements showed a thermal shrinkage of ca. 0.1% which is much lower compared to conventional PMMA molding (ca. 0.5%). However, considering the long cycle time together with the highly effective back pressure of the vario-thermal temperature control the low thermal shrinkage is something expectable.

### 2.3. Cell-Culture Trials for Validation Purposes

The experiments with hMSCs were performed with the approval of the Research Ethic Committee at Autónoma University of Madrid (UAM) and slightly modifying previously reported processes [18,19,20]. In short, the hMSCs were isolated from 1–2 mL of bone marrow-derived samples from healthy donors provided by Hospital La Princesa, the Jimenez–Díaz Foundation and Malaga University Biobank. Cells were isolated, and culture expanded following processes described also in previous research with minor modifications [18,19,20]. The preparation of the hMSCs-CM has been also presented in previous studies by our team linked to biofunctionalization of biomedical microdevices [21,22]. 

Once the conditioned medium is prepared, the multi-organ-on-chip biomedical microdevice is sterilized, washed and surface coated with the CM-hMSCs during 24 h in cell incubator, which is removed, just before seeding the microsystem with 10^3^ hMSCs drop by drop and using the six chamber inlets of the micro-injected polymeric replica used for the validation. Summarizing, the process follows previous research with some slight modifications [23,24]. The results from cell motility and colonization of the microsystems were determined by staining with crystal violet and by optical microscopy and are discussed in the following section. Cell viability was studied by means of real time microscopy with the cells showing a healthy behavior with expanded cytoskeletons and clearly moving. 

## 3. Results and Discussion

Once the computer-aided designs are improved taking account of the desired network of chambers and channels and once the master models additively generated by using laser stereolithography, the procedures previously described in the “Materials and Methods” section are applied to generate a metallic mold for micro-injection molding and to successfully produce a series of multi-scale organ-on-chip platforms using a transparent thermoplastic adequate for cell-culture studies, which are also performed. Relevant results, in connection with the validation of the different manufacturing steps, have been already presented in the “Materials and Methods” section for illustrating the different design and manufacturing processes. This section also discusses the results of the different processes for illustrating how the synergistic combination of technologies presented here finally works and how cells interact with the manufactured substrates. The potential of these synergistic technologies for the field of biomedical microdevices and microfluidic systems is also discussed. 

Starting with the laser processing of the master prototypes, Figure 2 shows the results from the laser material processing performed upon the master epoxy resin prototypes for generating the micro-pillars or connecting gates between vascular channels and organ chambers. Figure 2a provides a dimensional overview of chambers, channels and connecting gates, while Figure 2c provides details of the surface finish after laser material processing. The layer-by-layer generation of the master models leaves some slight horizontal marks in the walls of the channels and chambers, which can be appreciated in Figure 2a. However, the lateral walls of the opening gates, obtained by laser processing are extremely vertical(without the previously mentioned horizontal walls), which helps to highlight the viability of processing the epoxy resin of stereolithographic masters and the possibility of obtaining very precise cuts, in order to achieve the desired multi-scale geometries, as finally presented also in Figure 2c. 

The repeatability of the process is also noteworthy, as all the opening gates are 260 ± 5 µm and the remaining micro-pillars are 340 ± 5 µm. Such level of definition can be obtained with extremely high-precision additive manufacturing techniques, mainly resorting to two-photon polymerization (i.e., Photonic Professional system by NanoScribe GmbH), but the working field is normally limited to less than 1 mm^2^. Consequently, for organ-on-chip devices, in which relevant portions of tissues must be cultured and studied, the multi-scale combination of large field additive manufacturing techniques, for generating the overall chip structure, and the use of laser material processing, for defining the smaller details, proves more adequate with the current state of technology. Alternative approaches relying on the combination of multi-scale processes, such as mask-based lithography and high-precision direct laser writing (by two-photon polymerization), also allow for the generation of multi-scale organs-on-chips [25], although their productivity (parts manufactured per time unit) is still far from matching the performance of mass-produced systems by micro-injection molding, as presented here. Nevertheless, in terms of geometrical complexity and integration level [26], additive procedures remain unrivalled, so further synergies between processes may prove highly beneficial.

Once the master model is processed, the key challenge is linked to creating the metallic mold insert by nickel electroforming without damaging the master model and without losing the previously created details, which provide the desired functionality. Figure 3 shows the different steps for the metallization of master prototypes and the results of this process, which stands out for the surface quality achieved (see SEM images presented in Figure 4). The generated metallic insert, after dissolving the epoxy resin of the master model and final mechanical treatment using a milling process, is adjusted to create a mold cavity, which is machined and polished for improved flatness and mirror-like surface finish, as shown in Figure 5. The set of replicas is created by micro-injection molding (Figure 6), as previously detailed. Final productivity can be approximated to around 100–200 parts per hour, once the micro-injection process is adjusted and set to automated production. Multi-part molds are also possible, which would increase production, but the productivity achieved in present study is more than enough for our validation purposes and for illustrating the potentials of this synergistic combination of technologies. 

Improvements in terms of multi-scalability and precision may further be achieved by synergic nano-replication techniques, including coatings upon patterns obtained by nano-imprint lithography [27] and mold tools obtained by synergic combination of electron beam lithography, nano-imprint lithography, and ion beam etching [28].

**Figure 7:**
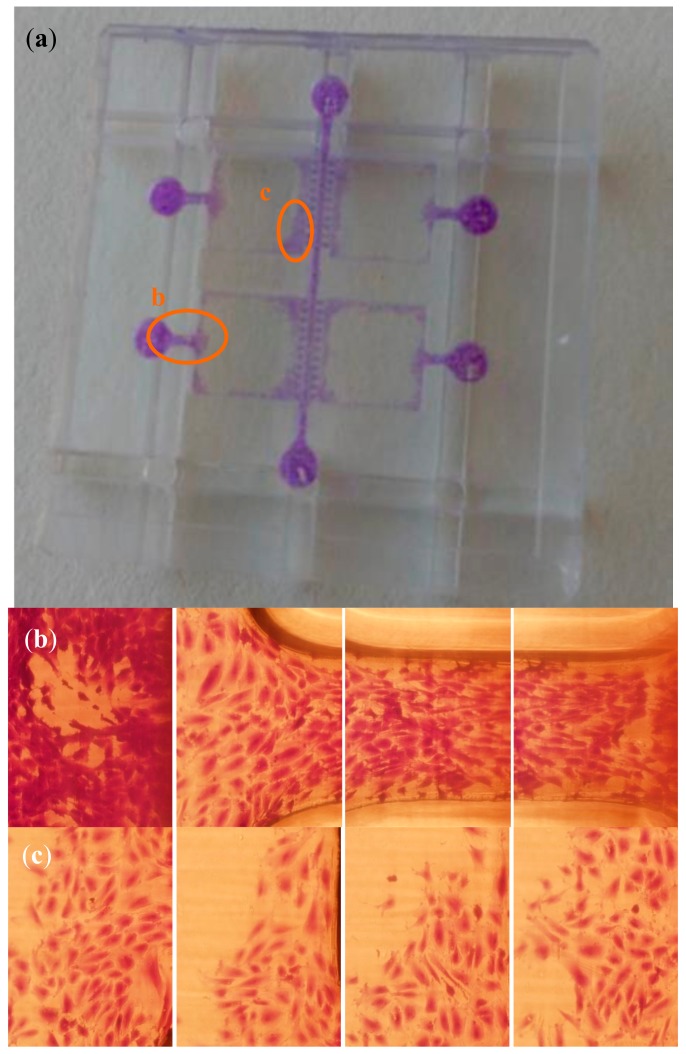
Results from human mesenchymal stem cells (hMSCs) colonizing the multi-chamber organ-on-chip platform obtained as mass-produced part by micro-injection molding: (**a**) Global view of the biomedical microdevice and magnified zones. (**b**) Sequences showing cells within an inlet well and along the channel connecting with an organ chamber. (**c**) Examples of cells colonizing the organ chambers. Cell staining performed with crystal violet.

Regarding cell-culture tests, the available results from hMSCs interacting within the multi-organ-on-chip biomedical microdevice are presented further on in the images of Figure 7. 

To this end one of the micro-injected replicas was exposed to CM produced by hMSC, as reported in early work, then seeded with cells and incubated with Dulbecco’s Modified Eagle’s Medium (DMEM) low glucose and 10% FBS during 48 h. The process is detailed in the “Materials and Methods” section. Figure 7a provides a global view of the biomedical microdevice and highlights the zones magnified in Figure 7b,c. Figure 7b shows cell adhesion to an inlet well and across a vascular channel entering or colonizing one of the organ chambers. Figure 7c shows cells colonizing an organ chamber after reaching it through the vascular channel and in between the micro-pillars although in other experiments the organ chamber may well be seeded independently (and even using different cell types for a true multi-organ-on-chip study). 

The cells are stained with crystal violet and, when analyzed with the microscope, show an expanded and healthy morphology. By careful visual inspection we can note that more than 100 cells/mm^2^ are present in such healthy conditions in several zones of the microsystem. Besides, it is important to highlight that hMSCs are adherent cells and that when they do not have enough metabolic energy to adhere they become spheroid, apoptotic and die in just a couple of hours. When hMSCs die, they appear floating in the cell-culture assays and disturb microscope imaging. In our tests we did not find a relevant number of dead and floating cells within the presented organ-on-chip platform and we could not appreciate any spheroidal cells, all of which helps to validate the adequate biological performance of the technologies, materials and methods used. As an example of the aforementioned unhealthy behavior of hMSCs, recent studies have demonstrated how the number of cells adhered is reduced if mitochondria have diminished electron transport and how this is directly linked to the progressive spheroidal configurations, apoptotic behavior, and final detachment [29]. Taking into consideration the biofunctionalization post-process performed by using the hMSCs-CM, we would like to mention that the coating improves hMSCs adhesion up to a 30–40%, when compared to the use of uncoated microsystems, as previous studies by our team have shown [12,23,24,26]. However, the manufactured layers can be also used for culturing other cell types and even hMSCs, just after micro-injection and polishing, without the need for a final biofunctionalization step. 

Even if we have worked with a single micro-structured device and cultured the cells within such platform just closing it with a transparent coverslip, which cannot be considered a completely functional organ-on-chip device, in which pumped fluids would support cells during their growth, proliferation and phenotype expression, we would like to mention that in many cases these preliminary cell-culture tests provide interesting information regarding cell-cell and cell-material interactions. In fact, the performed cell cultures have helped us to validate the viability of the micro-injected material for the industrial production of organ-on-chip platforms, or of their functional layers, and the possibility of working with these multi-chamber and multi-channel platforms, whose potential applications are varied. Future studies with these or similar micro-injected organ-on-chip platforms and cell-culture platforms will be linked to exploring the co-culture of different cell types and their communication, in accordance with recent progresses in cell-culture microsystems [1,2,30]. Synergies with other recent approaches to multi-scale mold insert fabrication, with which very special contact phenomena can be promoted, will be studied [31,32]. 

Finally, considering the issue of potential industrialization of the proposed approach, we envision that these set of technologies, with some additional supporting facilities, such as bonding and welding line, could be implemented in the form of a pilot line for the rapid engineering of lab- and organ-on-chip devices. The line would consist of: (1) a rapid prototyping technique working through additive photopolymerization, typically laser stereolithography or digital light processing, either in their industrial or low-cost modalities; (2) a laser micro-machining or ablation system for manufacturing the finer details and for performing surface modification and texturing for tuning the master models; (3) a electro-deposition system by galvanic bath for transforming the polymeric master models into metallic mold inserts and (4) an industrial micro-injection system with mold structures prepared for rapidly changing the inserts with the desired configurations of channels and chambers. In addition, supporting bonding and welding lines, together with fluid pumping systems, for the mounting and testing of complete microfluidic systems would be necessary. 

For simple lab- and organ-on-chip devices, in which the functional layer includes just micro-channels and chambers, the set of operations performed via laser stereolithography and subsequent laser ablation and metallization could be possibly replaced by a micro-milling machine working upon a metallic surface for directly generating the mold inserts. However, if complex geometries are involved, if aspect ratios above 1.5 are needed, or if details below 20 µm are needed, the combination of laser stereolithography and subsequent laser ablation performs better than micro-milling, especially when working with thermoset polymeric masters, whose machining by conventional subtractive methods is not so straightforward due to the higher heat-affected zones and debris generated. In fact, laser ablation reaches aspect ratios up to 50 for drilling and 10 for cutting and enables structuring materials with resolutions down to 1–10 µm, which result extremely demanding for micro-milling. In addition, laser material processing may be applied to obtain special surface textures, which can be applied to defining hydrophobic/hydrophilic transitions for controlling cell aggregation upon the surfaces of these types of microsystems. 

The main challenge for the proposed industrialization may be connected to training a community of researchers and technicians capable of managing this populated set of technologies, which requires vision, time, and resources. In any case, interesting alternatives may include: (a) the cooperation among delocalized research communities, as in fact we have shown in present study, or (b) the opening of large research infrastructures to external researchers through competitive calls, a possibility for which the KNMF-KIT constitutes a paradigmatic example. We expect these types of pilot lines and collaborative communities to impact at the bio-application level in this field of microfluidic devices by helping researchers to develop, in a straightforward way, sets of lab- and organ-on-chip devices, probably with a common framework or outer structure but with interchangeable functional layers, in which different patterns of channels, chambers and surface textures may help to mimic different organs and configurations of vascular networks and parenchymal tissues, towards the human-on-chip concept by integration of organ-on-chip microdevices.

## 4. Conclusions

In this paper, we have detailed and validated an innovative synergic combination of technologies for the design and rapid mass production of lab- and organ-on-chip platforms with complex geometries and multi-scale features for an improved biomimetic performance and with potential applications in the modeling and understanding of a varied cell-material and cell-to-cell interactions. The final biomedical microdevices obtained are transparent, which benefits imaging procedures; biologically apt, which enables cell-culture tests in adequate conditions; and geometrically repeatable due to the use of micro-injection molding, which promotes lab testing following systematic approaches. We aim to continue our research pursuing novel applications for of these multi-organ-on-chip devices and for the presented combination of production technologies, towards the industrial production of multi-scale biomedical microdevices. The described processes and geometries can be used, with some modifications, for many other fields, which may benefit from the use of microsystems with multi-scale features, including energy, transport and aerospace sectors, in which the use of micro-injected polymers may be of interest due to the low production cost and physical-chemical properties of these devices and materials.

## Figures and Tables

**Table 1:** Main parameters of the micro-injection molding replication experiments with PMMA (poly(methyl methacrylate)) as molding material.

Feature	Unit	Multi-Organ-Chip Micro-Injected Replica
Injection pressure	bar	1300
Injection speed	mm/s	33
Max. material temperature	°C	290
Tool temperature at injection	°C	130
Tool temperature at demolding	°C	65
Back pressure	bar	1050
Cycle time	s	≤300
